# Assessment and Diagnostic Standards of Childhood Apraxia of Speech in Turkish‐Speaking Children: A Scoping Review

**DOI:** 10.1111/1460-6984.70336

**Published:** 2026-09-15

**Authors:** Eddy C. H. Wong, Aşena Karamete, Ayşın Noyan‐Erbaş

**Affiliations:** ^1^ Academic Unit of Human Communication, Learning, and Development, Faculty of Education The University of Hong Kong Hong Kong, Hong Kong Special Administrative Region China; ^2^ Department of Speech and Language Therapy Faculty of Health Sciences Istanbul Atlas University Istanbul Türkiye; ^3^ Department of Speech and Language Therapy Faculty of Health Sciences Hacettepe University Ankara Türkiye

**Keywords:** assessment and diagnostic standards, childhood apraxia of speech, Turkish

## Abstract

**Background:**

Childhood apraxia of speech (CAS) is a motor speech disorder for which assessment and diagnostic standards have been largely informed by studies of English‐speaking populations. Evidence related to CAS in Turkish‐speaking children remains sparse, is published across both Turkish‐ and English‐language sources, and variable in diagnostic rigor.

**Aims:**

This scoping review aimed to map existing evidence on the assessment and diagnostic standards of CAS in Turkish‐speaking populations, with a focus on diagnostic approaches, assessment tasks, diagnostic quality, reported clinical features and gaps in the literature.

**Methods:**

The review followed the PRISMA Extension for Scoping Reviews guidelines. Systematic searches were conducted across thirteen English‐and Turkish‐language databases for peer‐reviewed articles published between 2007 and 2025. Studies reporting on at least one Turkish‐speaking child with CAS or suspected CAS were included. Extracted data included diagnostic methods, assessment tasks, reported clinical features and assessor information. Diagnostic quality was evaluated using an established rating framework and findings were synthesised narratively.

**Main Contribution:**

Five studies met the inclusion criteria, comprising 45 Turkish‐speaking children with CAS or suspected CAS. Diagnostic approaches ranged from reliance on general perceptual judgement to expert‐based evaluation and standardized assessment tools, with evidence of improved diagnostic quality over time. Assessment tasks sampled multiple domains, including single‐word production, connected speech, speech inconsistency, oral motor skills, diadochokinesis and prosody. Reported clinical features largely reflected language‐universal characteristics of CAS, with limited investigation of Turkish‐specific patterns.

**Conclusion:**

Although progress in CAS assessment for Turkish‐speaking children is evident, gaps remain in diagnostic consistency, psychometric evidence and language‐specific research, highlighting the need for further systematic investigation.

**WHAT THIS PAPER ADDS:**

*What is already known on this subject*

- Assessment and diagnosis of childhood apraxia of speech (CAS) are largely guided by evidence and assessment tools developed for English‐speaking populations. Research on CAS in Turkishspeaking children is limited, scattered across languages, and shows wide variability in diagnostic approaches and rigor, making it difficult to draw firm conclusions or guide clinical practice.

*What this study adds to the existing knowledge*

- This scoping review provides the first integrated synthesis of English‐ and Turkish‐language evidence on the assessment and diagnostic standards of CAS in Turkish‐speaking children. It demonstrates a gradual improvement in diagnostic quality over time and shows that reported clinical features largely mirror language‐universal characteristics of CAS, while Turkish‐specific manifestations remain under‐investigated.

*What are the clinical implications of this study?*

- Clinicians working with Turkish‐speaking children should adopt multi‐task assessment protocols that sample speech across varied contexts and use standardised tools, such as the DEMSS‐TR, to supplement expert perceptual judgement. The findings highlight the need for continued development, validation and clinician training in Turkish‐language CAS assessments to improve diagnostic consistency and accuracy.

## 1 Introduction

Childhood apraxia of speech (CAS) is a subtype of motor speech disorder with onset during speech development and is typically identified in childhood. It is characterised by the impairment of motor planning and programming of speech movements, resulting in speech sound and prosodic errors (American Speech‐Language‐Hearing Association [ASHA] 2007).

### 1.1 CAS Diagnostic Approach

The current gold standard for diagnosing CAS relies on expert perceptual judgement of speech characteristics elicited during structured assessment tasks and evaluated using pre‐defined clinical feature checklists (ASHA 2007; Murray et al. 2015; Strand et al. 2013). Although central to clinical practice, this perceptual approach is inherently subjective and recent work has highlighted the need to improve the reliability and consistency of clinician judgements across raters (Haley et al. 2026).

In response to this need, several structured clinical checklists have been developed, among which the Mayo Diagnostic Checklist is widely used. Although formal validity and reliability data for this checklist are limited, it conceptualises CAS as a constellation of perceptual speech features reflecting impairments in speech motor planning and programming, rather than a single pathognomonic marker (Grigos et al. 2024; Shriberg et al. 2011). The ten features include vowel distortions, voicing errors, phoneme distortions, articulatory groping, difficulty achieving initial articulatory configurations or transitioning movement gestures, intrusive schwa, increased difficulty with multisyllabic words, syllable segregation, slow speech rate and stress errors. Importantly, not all features traditionally associated with CAS are diagnostically discriminative. Strand (2020) identified a set of speech characteristics that are more likely to be diagnostically discriminative for CAS. These include impaired transitions between articulatory configurations, observable groping or trial‐and‐error behaviours, vowel distortions and prosodic disturbances such as lexical stress errors, equal stress or syllable segmentation. Additional discriminative features include inconsistent voicing errors, consonant distortions arising from blended manner features, intrusive schwa and inconsistency in the production of words or phrases across repeated trials. Importantly, these features are interpreted within a broader diagnostic framework rather than in isolation. According to the Mayo Diagnostic Checklist, a CAS diagnosis is supported when at least four out of the ten features are observed across a minimum of three speaking contexts (Grigos et al. 2024).

Given the importance of sampling speech across varied speaking contexts in the assessment of CAS, a group of researchers and expert clinicians proposed a minimal set of assessment data to support and confirm a CAS diagnosis in both research and clinical settings (McCabe et al. 2024). The suggested assessment domains include single‐word production, connected speech, repeated productions of single words and/or nonwords, oral motor evaluation, diadochokinesis (DDK) and patient‐reported outcomes (McCabe et al. 2024). A CAS specific dynamic assessment, the Dynamic Evaluation of Motor Speech Skills (DEMSS) was developed to supplement the diagnostic process for CAS (Strand and McCauley 2019). This dynamic approach offers advantages for differentiating motor speech impairment in children with severe speech sound disorders (SSDs), particularly in the observation of specific speech characteristics, judgements of severity and treatment planning (Strand and McCauley 2019). Given its satisfactory validity and reliability (Strand et al. 2013), the DEMSS has become an important component of CAS evaluation to support differential diagnosis, despite the non‐specific nature of its norms (broad, non‐specific cutoff scores).

### 1.2 CAS in Speakers of Languages Other Than English

The current understanding of CAS is primarily based on speakers of English and some Indo‐European languages, such as German and Dutch. Although CAS has been identified in a variety of languages (McCabe et al. 2024; Wong et al. 2026), understanding of CAS is limited in general and particularly in speakers of language other than English (LOTE). Wong and colleagues (2025; 2026) reported that most studies focused on the clinical presentation and comorbidities of CAS, with studies describing associated motor difficulties and speech‐language profiles or characteristics in several languages. Some investigations adapted English‐based assessments for use in different linguistic contexts, including the DEMSS and the Language‐Neutral Assessment of Motor Speech (Erlacher et al. 2025; Karamete and Toğram 2025). Other studies applied established intervention approaches in LOTE, such as integral stimulation, Dynamic Temporal and Tactile Cueing (DTTC), Rapid Syllable Transition (ReST) and the Prompts for Restructuring Oral Muscular Phonetic Targets (PROMPT). Further studies developed assessment tasks in specific languages (e.g., Arabic, Brazilian Portuguese and Cantonese) or proposed novel treatment approaches (e.g., Dutch, German and Ukrainian). In addition, a smaller body of research has examined the genetic and neural bases of CAS sought to identify biological features in speakers of LOTE (e.g., Italian and German). Finally, several studies explored SLPs’ perspectives on the clinical manifestation of CAS in under‐researched languages.

Among these studies of LOTE, language‐specific features were discussed. In Cantonese, children with CAS reported with lexical tone errors but not lexical stress errors, due to the prosodic differences between Cantonese and English (Wong et al. 2024; Wong, Wong, Velleman, et al. 2023). In French, children with CAS produced a minimal number of intrusive schwas, likely due to the high variability in schwa production across French dialects, where schwa is considered an optional vowel. Cluster reduction, however, was more prevalent than intrusive schwas (Meloni et al. 2020). In Hebrew, language‐specific manifestations of CAS were observed in relation to its rich morphological structure and consonant‐based root‐and‐pattern system, with reported difficulties in maintaining prosodic word structure, accurate affixation and segmental sequencing in morphologically complex forms, highlighting how CAS symptomatology may interact with the phonological and morphological demands of the target language (Tubi et al. 2024). These findings highlight the importance of examining CAS within the structural context of each language.

### 1.3 CAS in Turkish Speakers

Turkish is a language for which the understanding of CAS is still emerging, with a small but growing body of research addressing its phonological characteristics, assessment practices and clinical features. Turkish is an agglutinative language characterised by a relatively transparent phoneme–grapheme correspondence and a rich system of vowel harmony. Its phonological inventory includes sets of plosives, fricatives, affricates as well as nasals, laterals, approximants and flaps (e.g., [p, b, t, d, k, ɡ, f, v, s, ʃ, z, ʒ, tʃ, dʒ, h, l, j, r]). Consonant sequences across syllable boundaries are common in Turkish. In contrast, true consonant clusters in Turkish are restricted to word‐final position and follow a specific sonorant/fricative + stop or affricate pattern (e.g., /rk, rt, rp, nk, nt, nç, sk, st, lk, lp/); word‐initial clusters do not occur natively and are confined to loanwords (Topbaş 2006a). Stress in Turkish is generally predictable and typically falls on the final syllable of the prosodic word, although systematic exceptions occur in certain morphologically marked or lexical forms. The extensive use of derivational and inflectional suffixes leads to long and morphologically complex word forms (Ergenç 2002; Göksel and Kerslake 2005), placing demands on segmental and syllabic sequencing, prosodic planning and motor control (Maner et al. 2000). This linguistic characteristic might suggest differences in CAS features in Turkish versus other languages.

Recently, Karamete and Toğram (2025) adapted the DEMSS to Turkish (i.e., DEMSS‐TR) and reported satisfactory reliability, validity and diagnostic accuracy, providing an important foundation for the identification of CAS in Turkish‐speaking children with SSDs. Given that the original DEMSS was developed to supplement the diagnostic process for CAS by providing structured evidence across multiple speech tasks (Strand and McCauley 2019), its adaptation to Turkish raises the broader question of whether additional Turkish‐language assessment tools are available to support CAS diagnosis. This question is particularly relevant in light of recent recommendations that emphasize the need for a minimal set of assessment data drawn from varied speaking contexts to confirm CAS diagnosis in both research and clinical settings and to support future cross‐linguistic investigations (McCabe et al. 2024). Accordingly, a comprehensive understanding of existing Turkish‐language assessments is critical not only for strengthening local clinical and research practices, but also for informing assessment and diagnostic decision‐making in multilingual speakers, for whom evaluation in both/all languages is recommended when CAS or SSDs are suspected. As demonstrated by the scoping review of CAS in Chinese speakers (Wong et al. 2023), a comprehensive understanding of CAS in a specific language context requires the inclusion of both English‐language and local‐language publications to fully capture the understanding within a given language context.

### 1.4 Objectives

Thus, the objective of this scoping review was to map existing evidence on the assessment and diagnostic standards of CAS in Turkish‐speaking populations. Specifically, the review aimed to identify diagnostic approaches and assessment tasks used, examine diagnostic quality, summarize reported clinical features and highlight gaps in the current literature.

## 2 Methods

This scoping review followed the Preferred Reporting Items for Systematic Reviews and Meta‐Analyses Extension for Scoping Reviews guidelines (Tricco et al. 2018). It was informed by the Population–Concept–Context framework (Peters et al. 2020), with the population defined as individuals with CAS, the concept focusing on assessment and diagnostic standards, and the context limited to Turkish‐speaking populations.

### 2.1 Eligibility Criteria

The articles included in this review focused on speakers with CAS. The inclusion criteria were: (a) published in peer‐reviewed journals; (b) published between 2007 and2025; (c) written in English or Turkish; and (d) included at least one Turkish speaker diagnosed with CAS. Unpublished, non‐peer‐reviewed theses or dissertations were excluded. We selected the time frame based on the publication of the technical report on CAS in 2007 (ASHA 2007), which established a consensus definition of the disorder. Because diagnoses made before this publication often lacked consistency across studies, their reliability in relation to current diagnostic criteria is uncertain. Diagnostic quality was not used as an inclusion criterion; rather, it was evaluated as an outcome of interest to characterise the current state of CAS diagnosis in Turkish‐speaking children. Articles were excluded if they were (a) based on participants who speak a non‐Turkish language or are multilingual; (b) not available as full texts; or (c) review studies.

### 2.2 Information Sources and Search

A total of thirteen electronic databases were searched, including ten indexing English‐language literature and three indexing Turkish‐written literature. The English‐language databases were Cumulative Index to Nursing and Allied Health Literature (CINAHL) Plus, Embase, MEDLINE, ProQuest platform (including APA PsycInfo, Education Research Index, Medical Databases and ProQuest Dissertations & Theses Global: The Humanities and Social Sciences Collection), ScienceDirect, Scopus and Web of Science. The three Turkish‐language databases were DergiPark Akademik, TRDizin and Turk Medline.

Searches were conducted using the title, abstract and/or keyword fields depending on the capabilities of each database. A combination of keywords and truncations related to CAS and developmental speech disorders was used, for example: (“apraxi” OR “dyspraxi*”) AND (“develop*” OR “child*” OR “pediatri*” OR “paediatri*”) AND (“verbal” OR “speech”) AND (“Turkey” OR “Turkish”). Database‐specific limits were applied (e.g., publication type, peer review, language) and Boolean structures were adapted where search platforms imposed technical constraints (e.g., limits on maximum search terms in ScienceDirect). Appendix A presents an example of the search strategy for MEDLINE (an English database).

English‐language databases were searched by the first author on 11 December 2025 and Turkish‐language databases were searched by the second author on 12 December 2025. All search results were cross‐checked by the last author within one week as part of the verification process to ensure completeness and consistency across databases.

### 2.3 Selection of Sources of Evidence

Study selection involved title/abstract screening and subsequent full‐text eligibility assessment. Data extraction and diagnostic quality assessment were subsequently completed for all included studies.

Prior to the review process, calibration exercises were conducted following the recommendations of Wong et al. (2023) to establish a shared interpretation and consistent application of the study selection and diagnostic quality rating criteria among reviewers. For study selection calibration, five English‐language articles were randomly selected from the initial search results and independently evaluated by all three authors/reviewers. Full agreement regarding inclusion and exclusion decisions was achieved after the first round, with one article selected for full‐text review and the remaining four excluded.

The diagnostic quality rating scale proposed by Wong et al. (2023) (Table 1) was used to evaluate the diagnostic quality of CAS in the included articles. Given the different trajectories in the development of speech‐language pathology in China and Turkey, expertise was operationally defined for this review using both academic and clinical criteria. Academic expertise referred to individuals who were PhD holders or PhD candidates specialising in SSDs or motor speech disorders, whereas clinical expertise referred to individuals with at least five years of focused clinical experience working with children with SSD and/or CAS.

**TABLE 1:** Levels of quality of CAS diagnoses adapted from Wong et al. (2023).

Method	Level of quality	Example
Tests or methods with established validity and reliability	Ia	Tests or methods with established validity and reliability + objective data + expert perceptual judgement
	Ib	Tests or methods with established validity and reliability + objective data
	Ic	Tests or methods with established validity and reliability + expert perceptual judgement.
	Id	Tests or methods with established validity and reliability + non‐expert perceptual judgement
	Ie	Tests or methods with established validity and reliability only.
Objective data	IIa	Objective data + expert perceptual judgement
	IIb	Objective data + non‐expert perceptual judgement
	IIc	Objective data only
Expert perceptual judgement	IIIa	Expert perceptual judgement only
Non‐expert perceptual judgement	IIIb	Non‐expert perceptual judgement only
Unclear	IV	None

*Note*: Experts in this study are defined as people with academic expertise, who were PhD holders or PhD candidates specialising in speech sound disorders (SSD) or motor speech disorders, or with clinical expertise, who had at least five years of focused clinical experience working with children with SSD and/or CAS.

To calibrate the application of the diagnostic quality rating scale, one article randomly selected from the screened pool was independently evaluated by all three authors/reviewers in each round. The reviewers then discussed any discrepancies in quality ratings and refined the shared interpretation of the criteria. Consensus ratings were recorded as the final decisions for each article. These iterative rounds continued until full agreement on diagnostic quality ratings was achieved, which occurred after three rounds.

A separate calibration exercise was conducted for Turkish‐language articles, as only the second and last authors were fluent in Turkish. These two reviewers independently conducted data extraction and evaluated diagnostic quality for a randomly selected Turkish‐language article from the initial search results. A discussion followed to ensure consistency in interpretation and application of the rating criteria. Full agreement was achieved after one round.

**FIGURE 1:**
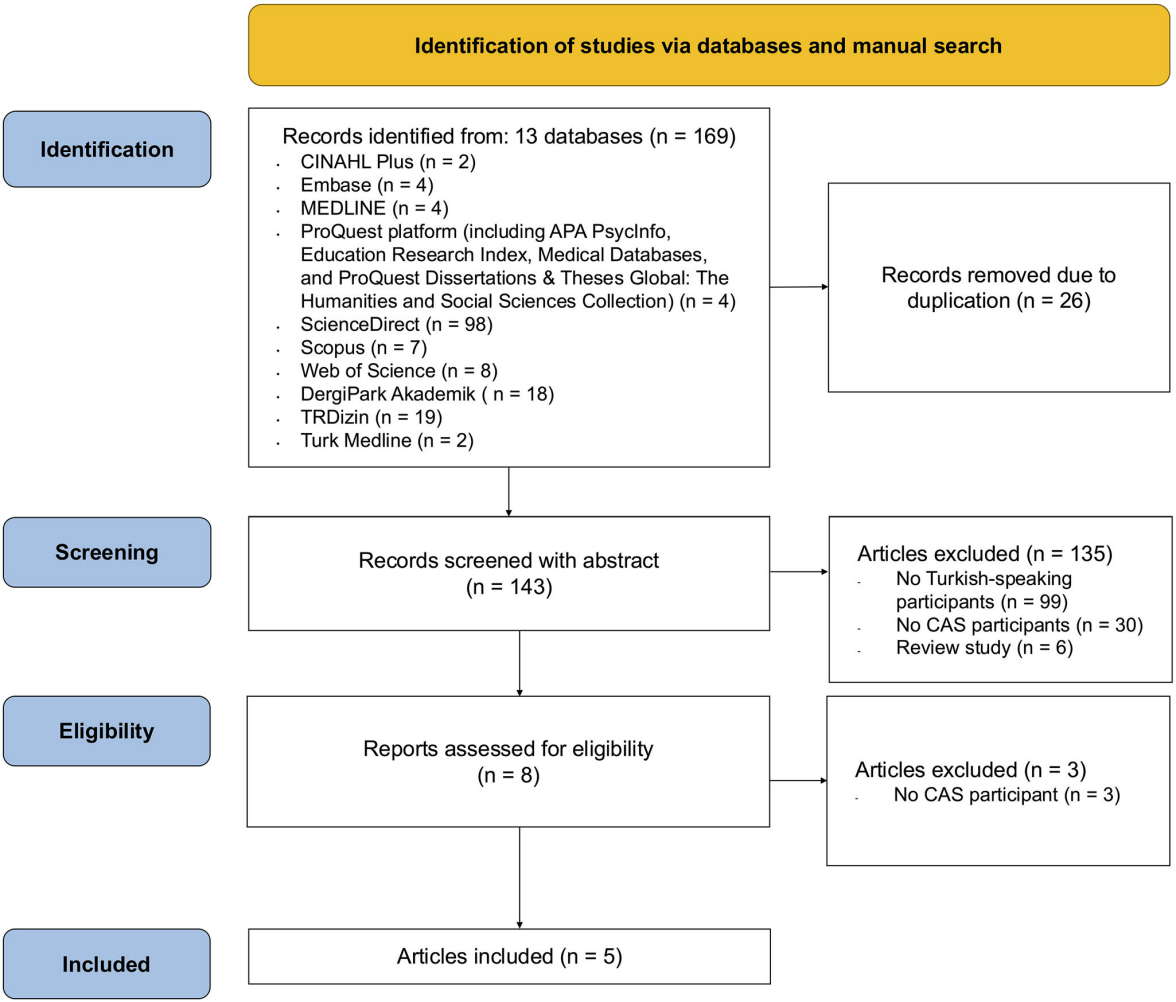
PRISMA flow diagram of the study selection process.

Following completion of the calibration exercises, the three authors/reviewers independently screened studies, extracted data and evaluated diagnostic quality. Disagreements were resolved through discussion and consensus. Figure 1 presents the PRISMA flow diagram. A total of 169 records were identified from ten databases and no additional records were located through manual reference searching. After removing 26 duplicates, 143 records were screened by title and abstract. Of these, 135 records were excluded for reasons such as the absence of Turkish‐speaking participants, no participants diagnosed with CAS or the study being a review article. Eight full‐text articles were assessed for eligibility and three articles (two Turkish‐language and one English‐language) were excluded because they did not include participants diagnosed with CAS. Ultimately, five studies met the inclusion criteria and were included.

### 2.4 Data Charting Process

Data extraction was completed using a structured Microsoft Excel spreadsheet. Extracted variables included (a) participants’ ages and genders, (b) initial diagnoses, (c) confirmation of CAS diagnosis, (d) assessment tasks, (e) diagnostic methods, (f) information about assessor and (g) diagnostic features and criteria. Reviewers independently extracted data, and discrepancies were resolved through discussion until consensus was reached.

### 2.5 Synthesis of Results

A narrative synthesis was used to summarize the extracted data, as the included studies varied widely in diagnostic methods, participant characteristics, assessment tasks, and reporting conventions. Extracted information was organized into descriptive categories, including participant demographics, initial and confirmed diagnoses, assessment procedures, diagnostic criteria, and reported speech characteristics.

Clinical and diagnostic features, if described, were grouped according to broad linguistic domains (e.g., segmental accuracy, prosody, phonotactics) as well as the consensus features of CAS described by ASHA (2007) and on the Mayo Diagnostic Checklist (Grigos et al. 2024; Shriberg et al. 2011). The quality‐of‐diagnosis rating derived from Wong et al. (2023) were used to differentiate studies with higher versus lower quality of diagnostic confirmation. In particular, the scale classified diagnostic processes into hierarchical levels based on three criteria: (a) use of reliable and valid tests or methods, (b) presence of objective data and (c) use of perceptual judgements. Higher ratings were assigned when more criteria were met and when judgements were made by clearly defined experts. The full set of levels and definitions used in the rating system is presented in Table 1.

## 3 Results

### 3.1 Participant and Study Information

Of the five included studies, three were written in English and two were written in Turkish. Table 2 presents the summary of the five included articles. A total of 45 Turkish‐speaking children with CAS or suspected CAS were reported, with ages ranging from 36 to 83 months (3; 0–6; 11). Two studies did not report participants’ ages or genders. In the remaining three studies, all participants were male, with a mean age of 66.7 ± 4.9 months and an age range of 63–83 months.

**TABLE 2:** Summary of the five included articles in this study.

Study	Design	CAS participant status	Number of CAS/sCAS participants	Age range (mean & SD)	Gender	Diagnostic methods	Assessor^^^	Diagnostic criteria	Quality of diagnosis
Polat and Logacev (2021)	Cross‐sectional comparative observational	Clinically diagnosed CAS	5	5;3–5;8 (5;5 ± 0.52)	5M	Perceptual judgement	SLP	Not specified	IV
Atila Çağlar and Noyan Erbaş (2022)	Single‐case intervention	Suspected CAS	1	6;11	1M	Perceptual judgement of 3 features	Non‐expert clinician	ASHA three consensus features	IIIb
İlman et al. (2024)	Cross‐sectional descriptive	CAS‐like features identified	3	3;0–6;11	Not specified	Test results	SLP	Below the cut‐off score	Ie
Arslan and Ünsal (2025)	Cross‐sectional comparative observational	Inferred within study	8	5;0–6;10 (5;5 ± 0.52)	8 M; 0F	Perceptual judgement of 11 features	Non‐expert clinician	Observed with >50% of the 11 features	IIIb
Karamete and Toğram (2025)	Methodological (psychometric validation)	Confirmed CAS / mild CAS / non‐CAS	28	3;0–6;11	Not specified	Perceptual judgement of 10 features from the Mayo Diagnostic Checklist	Expert clinician	≥ 4 out of 10 features observed in the assessment session	IIIa

Abbreviations: CAS, childhood apraxia of speech; F, female; M, male; sCAS, suspected childhood apraxia of speech; SLP, speech‐language pathologist.

^An expert SLP/clinician refer to either academic expertise that is, PhD holders or PhD candidates specialising in speech sound disorders (SSD) or motor speech disorders, or clinical expertise that is, at least five years of focused clinical experience working with children with SSD and/or CAS.

Study designs and aims varied among the included articles. Two studies were cross‐sectional comparative observational studies: One study compared differential diagnostic features of children with suspected CAS, phonological disorder and typical development (TD); examined CAS diagnostic criteria commonly reported in the English literature; and provided preliminary evidence to inform the development of a diagnostic checklist/test for CAS in Turkish‐speaking children (Polat and Logacev 2021). Another study examined aerodynamic and acoustic voice characteristics in children with suspected CAS and TD and determined whether these objective measures distinguish suspected CAS from children with TD (Arslan and Ünsal 2025). Karamete and Toğram (2025) reported a psychometric validation study, which aimed to adapt the DEMSS into Turkish and to established the reliability and validity of the DEMSS‐TR for the differential diagnosis of CAS. There was also a cross‐sectional descriptive study, which aimed to assess language, articulation and motor speech skills in young children with asthma, food allergy or hay fever and to identify the presence of developmental and motor speech risk indicators, including CAS (İlman et al. 2024). Finally, a single‐case intervention study aimed to describe the speech‐language profile of a Turkish‐speaking child with CAS and to examine the effectiveness of short‐term speech‐language intervention using an adapted version of the Kaufman Speech to Language Protocol (Ati̇la Çağlar and Noyan Erbaş 2022; Kaufman 2013).

### 3.2 CAS Diagnostic Confirmation and Classification

Across the five included studies, the levels of diagnostic confirmation of CAS varied substantially. Two studies included participants described as having CAS; however, the basis for the diagnosis differed. In Polat and Logacev (2021), children had been clinically diagnosed with CAS by SLPs prior to study inclusion, whereas Karamete and Toğram (2025) confirmed CAS diagnosis within the study using the Mayo Diagnostic Checklist (Grigos et al. 2024; Shriberg et al. 2011). Two studies investigated children with suspected CAS, where diagnostic status was inferred based on clinical judgement and checklist‐based criteria (Arslan and Ünsal 2025; Ati̇la Çağlar and Noyan Erbaş 2022). One study did not recruit participants based on CAS status but identified CAS‐like features through screening within a broader clinical population (İlman et al. 2024). Given the presence of underlying physical conditions in this population, the findings should be interpreted with caution regarding diagnostic specificity and the potential overlap between CAS and dysarthria.

### 3.3 Diagnostic Approaches

Varied diagnostic methods were adopted across studies, with the checklist approach being the most common method. In particular, Ati̇la Çağlar and Noyan Erbaş (2022) used the ASHA three consensus features (ASHA 2007). Arslan and Ünsal (2025) confirmed CAS diagnoses in participants using a checklist with eleven speech‐language features. Children who exhibited more than 50% of these features were confirmed with CAS. As mentioned, Karamete and Toğram (2025) used the Mayo Diagnostic Checklist and its suggested diagnostic criteria (four or more features observed in the assessment session) (Grigos et al. 2024; Shriberg et al. 2011) to confirm CAS in the participants. Another study (İlman et al. 2024) used the DEMSS‐TR and reported its psychometric properties for CAS diagnosis in Turkish speakers (i.e., DEMSS‐TR) as a screening tool to classify participants based on published cut‐off scores. The final study included participants with a prior CAS diagnosis; however, the specific diagnostic methods used to establish the diagnosis were not reported (Polat and Logacev 2021).

### 3.4 Diagnostic Quality

Diagnostic quality was evaluated using the rating scale proposed by Wong et al. (2023). Table 2 presents the ratings of each individual study. Overall, an improvement in diagnostic quality was observed over time, with more recent studies showing a shift from heavy reliance on clinicians’ perceptual judgements toward the use of standardized test‐based criteria, particularly following the successful development and application of the DEMSS‐TR.

### 3.5 Assessment Tasks

Table 3 presents the assessment tasks and reported clinical features observed in the CAS or suspected CAS participants. Across studies, assessment tasks spanned multiple speech and motor speech domains. Speech sound production was commonly assessed using a standardized, norm‐referenced single‐word articulation test, the Turkish Articulation and Phonology Test (Türkçe Sesletim‐Sesbilgisi Testi; SST) (Topbaş, 2006). The Turkish Early Language Development Test (Türkçe Erken Dil Gelişimi Testi; TEDİL) (Topbaş and Güven 2011) was also commonly used across studies to assess receptive and expressive language skills in the participants. A range of other assessments was reported, including cognitive screening; oral and speech motor assessment or screening, including DDK tasks; assessment of speech inconsistency across consecutive word production; prosodic assessment (sentence repetition with varied prosody or stress patterns); and a stimulability test.

**TABLE 3:** Summary of tasks and reported clinical features across five included studies.

Study.	Tasks or assessments.	Reported CAS characteristics and key findings.
Polat and Logacev (2021).	SST—Articulation Screening and the Auditory Discrimination Subtests. TEDİL.	Vowel errors, syllable stress errors, and intonation errors were most discriminative for CAS versus phonological disorder/typical development. Inconsistency and voicing errors were more frequent (not unique) in children with CAS. Isolated consonant production and spontaneous versus imitated differences were observed but discriminative power was limited. .
Atila Çağlar and Noyan Erbaş (2022).	SST—Articulation Screening Subtest. TEDİL. Assessment of speech inconsistency across consecutive production of 25 words. Stimulability test. Oral and speech motor assessment, including DDK tasks.	Pre‐treatment features included low intelligibility, limited phonetic–phonemic inventory, numerous developmental and non‐developmental phonological processes, ≥40% inconsistency, abnormal DDK, auditory‐perceptual prosodic differences, drooling and tongue thrust^^^. Treatment reduced inconsistency and improved stimulability.
İlman et al. (2024).	SST—Articulation Screening and the Auditory Discrimination Subtests. TEDİL. Ankara Early Development Inventory for cognitive skills screening. DEMSS‐TR.	In the allergy sample (*n* = 50), one child (2%) was identified with mild CAS, and two children (4%) were identified with CAS.
Arslan and Ünsal (2025).	SST—Articulation Screening Subtest. TEDİL. Assessment of speech inconsistency across consecutive production of 25 words. Prosodic assessment (sentence repetition with varied prosody or stress patterns). MPT. s/z ratio.	Checklist features frequently present in the sCAS group included ≥40% inconsistency, vowel errors, worse imitated than spontaneous speech, abnormal DDK, increased errors with utterance length and groping. Children with sCAS exhibited higher jitter, shimmer and noise‐to‐harmonics ratio. No group difference in fundamental frequency, MPT or s/z ratio.
Karamete and Toğram (2025).	SST—Articulation Screening Subtest. TEDİL. Ankara Early Development Inventory for cognitive skills screening. Anadolu University Oral Speech Mechanism Screening Form for oral structures, nonverbal oral motor imitation, DDK rates and dysarthria screening. DEMSS‐TR.	Inconsistent errors, vowel errors, prosodic disruption, reduced overall articulatory accuracy were observed during the dynamic assessment. Diagnostic cut‐offs (i.e., 375) showed high sensitivity and specificity for CAS diagnosis.

Abbreviations: CAS, childhood apraxia of speech; DDK, diadochokinetic rate; DEMSS‐TR, Dynamic Evaluation of Motor Speech Skills—Turkish Version (Karamete and Toğram 2025); MPT, maximum phonation time; sCAS, suspected childhood apraxia of speech; SST, Türkçe Sesletim‐Sesbilgisi Testi (Turkish Articulation and Phonology Test; TEDİL, Türkçe Erken Dil Gelişimi Testi (Turkish Early Language Development Test; Topbaş 2006b; Topbaş and Güven 2011).

^Drooling and tongue thrust are not considered core features of childhood apraxia of speech (CAS) and are more commonly associated with dysarthria; these findings should be interpreted with caution in differential diagnosis.

### 3.6 Reported CAS Characteristics

Across multiple included studies, a converging set of clinical features was reported in children with CAS or suspected CAS (Table 3). Inconsistent speech sound production across repeated word attempts was among the most frequently documented characteristics and was typically operationalised using consecutive productions of the same lexical items (e.g., repeated immediate productions of a single word before moving to the next items, rather than repeated productions of a word list). Vowel errors or distortions emerged as a prominent feature and were repeatedly identified as particularly informative for differentiating CAS from phonological disorder or TD. Prosodic disturbances, including atypical lexical stress and intonation patterns assessed perceptually or through structured sentence repetition tasks, were consistently highlighted in studies that directly examined suprasegmental features. Several studies also reported increased difficulty with longer or more complex utterances, although findings suggested that this feature alone had limited discriminative value. Motor speech–related characteristics, such as abnormal diadochokinetic performance, reduced speech motor control, groping, and difficulty initiating speech movements, were observed in studies incorporating oral–motor or dynamic speech assessments. Differences between spontaneous and imitated speech, with poorer performance under imitation, were noted in children with suspected CAS. One study further reported atypical acoustic voice characteristics, including higher noise‐to‐harmonics ratio, jitter, and shimmer. Notably, Atila Çağlar and Noyan Erbaş (2022) reported additional features such as drooling and tongue thrust; however, these characteristics are more commonly associated with dysarthria and are not considered core features of CAS, highlighting the need for careful differential diagnosis. Maximum phonation time and s/z ratio did not differentiate groups. Finally, dynamic assessment using the DEMSS‐TR identified inconsistent errors, vowel errors, prosodic disruption and reduced overall articulatory accuracy. The total score derived from measures of consistency, articulatory accuracy, vowel accuracy, and prosodic accuracy demonstrated strong diagnostic sensitivity using established cut‐off scores.

## 4 Discussion

This scoping review provides a comprehensive synthesis of English‐ and Turkish‐language literature on the assessment and diagnostic standards of CAS in Turkish‐speaking populations. By examining diagnostic approaches, assessment tasks, diagnostic quality and reported clinical features, these findings highlight both areas of convergence with the broader CAS literature and important gaps specific to the Turkish context.

### 4.1 Diagnostic Quality of CAS in Turkish‐Speaking Children

The diagnostic approach to CAS in Turkish‐speaking children has evolved over time, reflecting an overall improvement in diagnostic quality. Earlier studies primarily relied on general perceptual judgement of speech characteristics, often without clearly specified diagnostic criteria or standardised procedures (Level IIIb and V) (Arslan and Ünsal 2025; Ati̇la Çağlar and Noyan Erbaş 2022; Polat and Logacev 2021). More recent research, however, has emphasized expert perceptual judgement grounded in established CAS feature sets (Level IIIa) (Karamete and Toğram 2025) and increasingly incorporated standardised, psychometrically evaluated assessment tools (Level Ie) (İlman et al. 2024).

The adaptation and validation of the DEMSS‐TR represents a notable advancement in this regard, as it introduces a structured and empirically supported instrument to supplement clinical judgement. This shift toward combining expert perceptual judgement with valid and reliable assessment tools aligns with contemporary recommendations for improving diagnostic accuracy in Turkish speakers with CAS and reduces reliance on subjective impressions alone. Consistent with the original English DEMSS (Strand and McCauley 2019), the DEMSS‐TR employs broad, non‐age‐specific cut‐off scores rather than age‐based norms; for example, a total score cut‐off of 375 yielded 93% sensitivity and 96% specificity for distinguishing CAS from non‐CAS across the 3;0–6;11 age range studied (Karamete and Toğram 2025). Non‐age‐specific cut‐off scores were adopted because the DEMSS‐TR, consistent with the original DEMSS is a criterion‐referenced rather than norm‐referenced instrument. It is designed to characterise the severity and pattern of CAS‐consistent features within a population already identified with SSD, rather than to benchmark performance against typically developing, age‐matched peers. Because CAS reflects a speech motor planning and programming deficit rather than a developmental pattern expected to resolve with age within the 3; 0–6; 11 ranges studied, symptom severity, rather than age or gender, was considered the diagnostically relevant dimension. Therefore, a single clinical threshold was considered appropriate across the sample.

As a dynamic assessment, the DEMSS and its Turkish adaptation place substantial demands on examiner expertise, and assessment outcomes may be influenced by the clinician's level of training and experience with children who have SSDs or CAS. Although both the DEMSS and DEMSS‐TR have demonstrated satisfactory inter‐rater reliability, the literature consistently emphasises the importance of pre‐administration training to ensure consistency in administration, scoring and diagnostic decision‐making.

### 4.2 Available Turkish Assessments

Given the supplementary nature of the DEMSS and the recommendation to avoid reliance on a single measure for CAS diagnosis (Preston et al. 2021), consideration of a comprehensive assessment protocol that samples multiple speaking contexts and domains remains essential. This approach is consistent with recommendations from a group of CAS researchers and expert clinicians, who proposed a minimal set of assessment data to support accurate CAS diagnosis in both clinical and research settings (McCabe et al. 2024).

Findings from the present review indicate that a range of assessment tasks has been used in Turkish‐language studies to support the evaluation of CAS, including single‐word production, connected speech sampling, repeated productions of single words and/or nonwords, oral motor evaluation and DDK tasks. For example, the Turkish Articulation and Phonology Test and its Articulation Screening Subtest (Topbaş 2006b) allow examination of speech sound production in Turkish and comparison with available normative data. Inconsistency measures used by Atilla Çağlar and Noyan Erbaş (2022) and Arslan and Ünsal (2025) function as assessments of repeated productions of single words, requiring consecutive repetitions of a standardised list of 25 Turkish words.

In addition, oral motor evaluation and DDK tasks were incorporated through the Anadolu University Oral Speech Mechanism Screening, as used by Karamete and Toğram (2025). This screening tool allows for the evaluation of oral structures, nonverbal oral motor imitation skills, speech motor abilities (including DDK rates) and the presence of dysarthria. Prosodic skills were also examined in some studies using sentence repetition tasks with varied prosodic or stress patterns (Arslan and Ünsal 2025), providing insights into prosodic control in Turkish‐speaking children. Collectively, these assessments and tasks have the potential to form a comprehensive CAS assessment protocol that aligns with international guidelines for CAS assessment and differential diagnosis.

Although the present review did not include patient‐reported outcome measures specifically developed for Turkish‐speaking children with CAS, a Turkish version of the Intelligibility in Context Scale (McLeod et al. 2012; Topbaş 2012), with established validity and reliability (Şanlı 2022), is available and may serve as a preliminary proxy for caregiver‐reported intelligibility in everyday communication. When considered collectively, the available tasks and tools are capable of supporting differential diagnosis between CAS and other SSDs. The availability of the DEMSS‐TR further strengthens the assessment landscape by providing a standardized dynamic assessment that systematically samples motor speech performance across varying syllable shapes and phonetic contexts. However, few studies explicitly justified task selection with reference to minimal data set recommendations, and the extent to which existing Turkish assessments collectively fulfil these criteria remain unclear. Future research would benefit from explicitly mapping Turkish assessment tools onto established diagnostic frameworks to ensure comprehensive, theory‐driven CAS evaluations in both research and clinical practice.

### 4.3 Clinical Features of CAS in Turkish Speakers

Across the reviewed studies, reported clinical features of CAS in Turkish‐speaking children largely reflect language‐universal characteristics commonly described in the broader CAS literature, such as inconsistent speech errors, disrupted coarticulatory transitions, syllable segmentation and prosodic disturbances (Wong et al. 2020). In addition, some voice‐related or phonatory characteristics have been noted, such as exhibition of higher jitter, shimmer and noise‐to‐harmonics ratio (Arslan and Ünsal 2025). Together, these findings suggest that CAS in Turkish‐speaking children may involve multiple interacting components of the speech motor system, including segmental, prosodic, and phonatory domains.

These findings suggest that many core features of CAS observed in Turkish speakers are likely generalisable to children with CAS across languages. At the same time, there is a notable lack of studies explicitly designed to investigate language‐specific manifestations of CAS in Turkish. Given Turkish's agglutinative morphology, predictable stress patterns and frequent use of morphologically complex word forms, potential language‐specific markers may include difficulties with affix sequencing, maintenance of prosodic word structure across long utterances and increased breakdowns at morpheme boundaries. The scarcity of targeted investigations limits firm conclusions in this area and highlights an important gap in the literature. Systematic, hypothesis‐driven studies are therefore needed to clarify how the structural properties of Turkish may shape the surface manifestations of CAS and to determine which features are truly language‐specific versus cross‐linguistically robust.

### 4.4 Limitations and Future Investigations

Several limitations of the present scoping review should be acknowledged. First, the available evidence basis on CAS in Turkish‐speaking children remains relatively small and heterogeneous, with many studies relying on small sample sizes, descriptive designs or lower levels of evidence. As a result, conclusions regarding diagnostic quality, assessment practices and reported clinical features should be interpreted cautiously. In addition, variability in how CAS was defined and operationalized across studies, particularly in earlier work that relied primarily on general perceptual judgement and included both children with confirmed CAS and those with suspected CAS, limited direct comparability and synthesis across studies.

Second, although the review identified a range of assessment tasks used in Turkish‐language studies, relatively few investigations explicitly mapped task selection onto established minimal data set recommendations for CAS diagnosis. Psychometric evidence for several commonly used assessment tasks also remains limited, restricting conclusions regarding their diagnostic sensitivity and specificity, particularly for CAS. The absence of validated patient‐reported outcome measures specifically designed for Turkish‐speaking children with CAS represents an additional gap, despite the availability of translated tools such as the Intelligibility in Context Scale.

Building on these limitations, several directions for future research are warranted. There is a clear need for higher‐quality, hypothesis‐driven studies that employ standardised diagnostic frameworks and clearly defined CAS criteria in Turkish‐speaking populations. Future investigations should explicitly examine how existing Turkish assessment tools align with recommended minimal data sets and identify areas where additional task development or validation is required. In particular, studies designed to establish the psychometric properties of Turkish‐language assessment measures, including reliability, validity, and diagnostic accuracy, would substantially strengthen both clinical and research practices.

In addition, existing Turkish assessments and tasks rely heavily on perceptual evaluation, such as phonetic transcription and judgements regarding the presence or absence of specific speech characteristics, including within standardised tools such as the DEMSS‐TR. While perceptual judgement remains central to CAS diagnosis, this reliance introduces a degree of subjectivity. Future research could therefore focus on developing more objective diagnostic indicators, including empirical cutoff values for existing measures and quantitative indices to support CAS identification. The acoustic measures of voice‐related characteristics proposed by Arslan and Ünsal (2025) offer a promising foundation for further development in this area.

Moreover, targeted investigations of language‐specific manifestations of CAS in Turkish are needed. Given the agglutinative morphology and predictable stress system of Turkish, future studies should systematically explore whether features such as affix sequencing difficulties, breakdowns in prosodic organisation across morphologically complex words, or errors at morpheme boundaries may serve as language‐specific markers of CAS. The current scarcity of such investigations limits firm conclusions and highlights an important direction for future research.

The present scoping review focused on assessment and diagnosis and did not address treatment outcomes. Future investigations evaluating the efficacy and effectiveness of intervention approaches for Turkish‐speaking children with CAS are therefore needed to inform evidence‐based clinical practice. Finally, research examining CAS assessment in bilingual and multilingual speakers that include Turkish as one of the languages would be especially valuable; as such work would inform both cross‐linguistic theory and clinical decision‐making in increasingly diverse populations.

## 5 Conclusion

This scoping review synthesised existing English‐ and Turkish‐language evidence on the assessment and diagnostic standards of CAS in Turkish‐speaking children. The findings indicate gradual progress in diagnostic practices, with a shift from reliance on general perceptual judgement toward the use of expert‐based evaluation and standardised, psychometrically supported tools. At the same time, substantial variability remains in diagnostic approaches, assessment task selection, and diagnostic quality across studies, reflecting the emerging nature of CAS research in Turkish‐speaking populations.

Across the reviewed literature, assessment practices in Turkish generally align with internationally recommended domains for CAS evaluation; however, explicit alignment with minimal data set recommendations and comprehensive psychometric validation of Turkish‐language tools remain limited. Reported clinical features largely mirror language‐universal characteristics of CAS, while evidence for Turkish‐specific manifestations remains sparse and underexplored. These gaps constrain diagnostic consistency and limit cross‐linguistic comparability.

Overall, this review underscores the importance of integrating both English‐ and Turkish‐language literature to achieve a comprehensive understanding of CAS in a specific language context. Future research should prioritise standardised diagnostic frameworks, systematic validation of assessment tools, targeted investigation of language‐specific features and examination of CAS assessment in bilingual and multilingual speakers. Such efforts are essential for strengthening evidence‐based, culturally and linguistically appropriate clinical practice for Turkish‐speaking children with CAS.

## Funding

The authors have nothing to report.

## Ethics Statement

Ethics approval was not required for this review study as it did not involve human participants or the collection of new data.

## Consent

Patient consent was not required for this study.

## Conflicts of Interest

The authors declare no conflicts of interest.

## Data Availability

This study is based on a review of previously published literature. No new datasets were generated or analysed, and all data supporting the findings of this study are included within the article and its references.
